# Sphingosine kinase 1 expression enhances colon tumor growth

**DOI:** 10.1186/s12967-017-1220-x

**Published:** 2017-06-06

**Authors:** Hideki Furuya, Yoshiko Shimizu, Paulette M. Tamashiro, Kayoko Iino, Jacek Bielawski, Owen T. M. Chan, Ian Pagano, Toshihiko Kawamori

**Affiliations:** 1 Cancer Biology Program, University of Hawaii Cancer Center, 701 Ilalo Street, Honolulu, HI 96813 USA; 2 Clinical and Translational Research Program, University of Hawaii Cancer Center, 701 Ilalo Street, Honolulu, HI 96813 USA; 3 Department of Molecular Biosciences and Bioengineering, University of Hawaii at Manoa, Honolulu, HI 96822 USA; 4 Department of Biochemistry and Molecular Biology, Medical University of South Carolina, Charleston, SC 29425 USA; 5 Cancer Prevention in Pacific Program, University of Hawaii Cancer Center, Honolulu, HI 96813 USA; 6 Shonan Medical Clinic, 6F Sakurabashi Plaza Build., 1-4-12 Sonezakishinnchi, Kita-ku, Osaka, 530-0002 Japan

**Keywords:** Sphingolipids, SphK1 KO mouse, Xenograft, SphK1 overexpression mouse, HT-29 cells

## Abstract

**Background:**

Accumulating evidence suggests that sphingosine kinase 1 (SphK1)/sphingosine 1-phosphate pathway plays a pivotal role in colon carcinogenesis.

**Methods:**

To further support the evidence, we investigated the effects of SphK1 using three separate animal models: SphK1 knockout mice, SphK1 overexpressing transgenic mice, and SphK1 overexpression in human colon cancer xenografts. Using azoxymethane (AOM, colon carcinogen), we analyzed colon tumor development in SphK1 KO and SphK1 overexpression in intestinal epithelial cells regulated by a tet-on system. Then, we analyzed subcutaneous tumor growth using xenografts of HT-29 human colon cancer cell. Finally, immunohistochemical analyses for SphK1 and COX-2 were performed on human colon cancer tissue microarray.

**Results:**

SphK1 KO mice, compared to wild-type mice, demonstrated a significant inhibition in colon cancer development induced by AOM (58.6% vs. 96.4%, respectively, *P* < 0.005). Tumor multiplicity (1.00 vs. 1.64 per colon, respectively, *P* < 0.05) and tumor volume (14.82 mm^3^ vs. 29.10 mm^3^, *P* < 0.05) were both significantly reduced in SphK1 KO mice compared to wild-type mice. Next, SphK1 overexpression in HT-29 enhanced tumor growth as compared to GFP control in nude mice (229.5 mm^3^ vs. 90.9 mm^3^, respectively, *P* < 0.05). Furthermore, overexpression of SphK1 in intestinal epithelial cells significantly enhances AOM-induced colon tumor formation (*P* < 0.05). Lastly, SphK1 and COX-2 intensity tended to reduce overall survival of late stage colon cancer patients.

**Conclusions:**

SphK1 expression regulates the early stage of colon carcinogenesis and tumor growth, thus inhibition of SphK1 may be an effective strategy for colon cancer chemoprevention.

**Electronic supplementary material:**

The online version of this article (doi:10.1186/s12967-017-1220-x) contains supplementary material, which is available to authorized users.

## Background

Colorectal cancer is the second leading cause of all cancer-related deaths in the US with a reported 50,260 annual deaths [1]. Despite aggressive screening guidelines for early detection and detailed knowledge of several critical events underlying the pathogenesis of cancer, colorectal cancer continues to be a major health concern in developed countries.

Sphingolipids, especially ceramide, sphingosine, ceramide-1-phosphate and sphingosine-1-phosphate (S1P), play key roles as regulatory molecules in cancer development [2]. S1P promotes cell proliferation and survival, and regulates angiogenesis, whereas sphingosine and ceramide inhibit cell proliferation and stimulate apoptosis. Sphingosine kinase (SphK) phosphorylates sphingosine to form S1P and is a critical regulator of sphingolipid-mediated functions. Two mammalian isozymes, SphK1 and SphK2, have been characterized [3]. An inducible SphK1 is activated by numerous growth factors and cytokines, and recent studies have revealed that the SphK1/S1P pathway regulates the COX-2/prostaglandin E_2_ (PGE_2_) pathway [4–8]. Furthermore, SphK1 knockout (KO) mice demonstrated a lower incidence of colon cancer development in an inflammation-related colon carcinogenesis model [5]. On the other hand, the effect of SphK2 in colon carcinogenesis is still under discussion. For example, previous study demonstrated that SphK2 deficiency has no effect on colon carcinogenesis [5], whereas SphK2 inhibitor ABC294640 has inhibitory effects on acute colitis and colitis-driven colon carcinogenesis [9, 10]. Taken together, the SphK1/S1P pathway may play a pivotal role in colon carcinogenesis. However, the mechanisms by which this pathway mediates colon carcinogenesis are yet to be elucidated.

In this study, we investigated effects of SphK1 expression in intestinal epithelial cells on colon carcinogenesis. First, we demonstrated that lack of SphK1 expression significantly inhibited AOM-induced colon tumor development using SphK1 KO mice. Then SphK1 overexpression in human colon cancer cells (HT-29) enhanced tumor development in a subcutaneous xenograft model. Moreover, we demonstrated that overexpression of SphK1 in intestinal epithelial cells significantly enhanced colon tumor development using double transgenic mice with doxycycline treatment. Lastly, we demonstrated that SphK1 and COX-2 intensity tended to reduce overall survival of late stage colon cancer patients.

## Methods

### Mice

Three independent, yet complementary, animal experiments were sequentially conducted. Mice were housed and handled in the animal and veterinary service program in University of Hawaii (UH). Mice were maintained under controlled conditions of humidity (50 ± 10%), light (12/12 h light/dark cycle) and temperature (23 ± 2 °C). AOM was obtained from the NCI Chemical Carcinogen Reference Standard Repository (Bethesda, MD). In double Tg mice experiments, all animals were provided with Doxycycline diet (Dox, 200 mg/kg, S3888, Bio Serv, Frenchtown, NJ, USA).

### SphK1 KO mice—AOM-induced colon carcinogenesis model

SphK1 homozygous KO mice of the 129SV-C57BL/6 background, a kind gift from Dr. Richard L. Proia [National Institute of Diabetes and Digestive and Kidney Diseases (NIDDK)/NIH, Bethesda, MD, USA], were backcrossed to C57BL/6 wild-type (WT) mice (purchased from Charles River Laboratories, Wilmington, MA, USA) at least 10 times [11]. Male mice at 6 weeks of age were intraperitoneally injected with either vehicle or AOM (10 mg/kg body weight) once a week for 6 weeks. All mice were provided with food and tap water ad libitum. All animals were sacrificed at 50 weeks after the last AOM administration. Blood samples were taken through heart puncture after sacrifice and snapped frozen using liquid nitrogen and stored in −80 °C for sphingolipid profile analysis. After laparotomy, the entire stomach and intestines were resected and opened longitudinally and the contents were flushed with normal saline. Using a dissection microscope, large intestinal tumors were noted grossly for their location, number and size. The length (*L*), width (*W*) and depth (*D*) of each tumor were measured with calipers and tumor volume (*V*) was calculated using the formula *V* = *L*∙*W*∙*D*∙π/*6* [12]. Colon tumors and normal tissues were fixed in 10% buffered formalin and embedded in paraffin blocks for histological evaluation. Diagnosis of intestinal tumors using hematoxylin and eosin stained sections was performed according to classification of Krutovskikh [13].

### SphK1 overexpression in subcutaneous xenograft model

Female nude mice were purchased from Charles River Laboratories. HT-29 human colon adenocarcinoma cell line (ATCC, Rockville, MD) was maintained in Dulbecco’s Modified Eagle’s Medium (DMEM, Invitrogen, CA) plus 10% Fetal Bovine Serum (FBS, Invitrogen) and 1% Penicillin/Streptomycin (Invitrogen) in a humidified 5% CO_2_ incubator at 37 °C. The hSphK1-GFP overexpressing vector was constructed according to the previous study [14]. HT-29 cells were transfected with hSphK1-GFP vector or GFP empty vector using Lipofectamine 2000 (Invitrogen) according to the manufacturer’s protocol. Stable transfectants were selected and maintained using G418 (1 mg/ml, Invitrogen) in media. One million GFP stably transfected HT-29 cells (GFP-HT-29 cells) or hSphK1-GFP stably transfected HT-29 cells (hSphK1-HT-29 cells) were injected subcutaneously into six female nude mice in each group. Mice were monitored every 3 days for 6 weeks and data including body weight and tumor size were collected. Complete laparotomy was performed to investigate metastasis and all tumor tissues were collected frozen in liquid nitrogen and stored at −80 °C for analysis of sphingolipid profile.

### SphK1 overexpression mice—AOM-induced colon carcinogenesis model

To create transgenic mice with SphK1 expression in intestinal epithelial cells, we selected a tet-on system, a binary transgenic system in which SphK1 expression is dependent on the activity of an inducible transcriptional activator; reverse tetracycline-regulated trans-activator (rtTA). Transgenic mice with *Fabpl* ^*4x at* −*132*^ */rtTA* that expresses in intestinal epithelial cells have been already established [15] and a tet-on system controls SphK1 expression with doxycycline treatment. We cloned hSphK1 [14] into a pcDNA4/TO vector (Invitrogen). We confirmed that double transfectants with pcDNA4/TO-hSphK1 and pcDNA6/TR induce hSphK1 protein and activity in MCF-7 cells (Additional file 1: Figure S1A, B, respectively). The tetO-SphK1 transgene construct was excised from pcDNA4/TO-hSphK1 plasmids using Xmnl, Mfel, and Bst1107 l, purified, and microinjected into FVB/N mouse oocytes and established tetO-hSphK1 transgenic mice (Additional file 1: Figure S2). Transgenic mice containing *Fabpl* ^*4x at* −*132*^ */rtTA* (rtTA mice) obtained from the NCI-Frederick Mouse Models of Human Cancers Consortium (MMHCC) Repository were established. We confirmed double transgenic mice (double Tg) after mating tetO-hSphK1 mice and rtTA mice. Genotypes of SphK1 KO mice, tetO-hSphK1 Tg mice, rtTA mice and double Tg mice were determined by polymerase chain reaction (PCR) analysis of genomic DNA from tail biopsies (Additional file 1: Table S1) [11, 15].

### Sphingolipid profile

Levels of serum S1P in blood samples were analyzed by sphingolipid profiling using tandem mass spectrometry with positive-mode electrospray ionization in the Medical University of South Carolina (MUSC) Lipidomics Core Facility, as described previously [6]. Results of serum samples were expressed as picomoles of S1P per 100 μl of blood and per mg protein, respectively.

### Human colon cancer samples and immunohistochemical staining

Human colon cancer tissue microarray slide (TMA) was purchased from US Biolab (cat. # Col180Sur-03, Gaithersburg, MD). The TMA slide includes 90 cases of colon adenocarcinomas and matched normal adjacent tissues. Demographic, clinical and pathologic characteristics of the 90 cases comprising the study cohort are illustrated in Table 1. Immunohistochemical staining was performed using standard protocols. TMAs were deparaffinized in xylene and rehydrated using graded percentages of ethanol followed by antigen retrieval with citric acid buffer (pH 6.0, 95 °C for 20 min). The slides were treated with 3% hydrogen peroxide in water to block endogenous peroxidase activity. Staining for SphK1 and COX-2 was conducted using rabbit anti-human SphK1 antibody (AP7237c; 1:100 dilution in blocking buffer, Abgent, San Diego, CA) and Rabbit anti-COX-2 antibody (CRM 306 A; 1:100 dilution in blocking buffer, Biocare Medical, Concord, CA), respectively. Biotin-labeled horse anti-rabbit IgG (2 µg/ml in blocking buffer, Vector Laboratories) was used as the secondary antibody. Immunoreactive signals were amplified by formation of avidin–biotin peroxidase complexes and visualized using 3,3′-diaminobenzidine (DAB). Nuclear counterstaining was conducted with hematoxylin. Immunohistochemical expression was measured by a board-certified pathologist (OC) by calculating scores for SphK1 or COX-2 immunostaining in colorectal samples: (0) negative; (1) weak staining in <50% of cells; (2) moderate staining in >50% of cells; (3) strong staining in >50% of cells.

**Table 1:** Demographic, clinical and pathologic characteristics of 90 subjects comprising the study cohort

Features	Patients (%) N = 90
Age (years)	
<65	54 (60%)
>65	35 (39%)
Unavailable	1 (1%)
Sex	
Female	38 (42%)
Male	51 (57%)
Unavailable	1 (1%)
Survival status	
Alive	48 (53%)
Dead	42 (47%)
Tumor grade	
G1	21 (23%)
G2	48 (53%)
G3	21 (23%)
Tumor stage	
T1	1 (1%)
T2	4 (4%)
T3	61 (68%)
T4	24 (27%)
Follow-up (month)	
Median	57

### Statistical analysis

In an AOM-induced colon carcinogenesis model, tumor incidence, expressed as the percentage of tumor-bearing animals, was analyzed with the Chi square test. Tumor multiplicity, expressed as the number of tumors per mouse, and tumor volume (mm^3^) were analyzed with the unpaired Student’s *t* test (comparing SphK1 KO mice and WT mice, or SphK1 double Tg with and without doxycycline diet). In a xenograft model, tumor sizes and levels of S1P (pmol/mg protein) were analyzed with the unpaired Student’s *t* test between SphK1 overexpression and GFP control HT-29 cells at indicated time points. Wilcoxon matched-pairs signed rank tests compared overexpression in cancer tissues and adjacent normal tissues. The GraphPad Prism version 6.0 software (GraphPad Software, La Jolla California USA, http://www.graphpad.com) performed all analyses. Differences were considered statistically significant at *P* < 0.05.

## Results

### SphK1 KO mice significantly reduce AOM-induced colon carcinogenesis

**Fig. 1:**
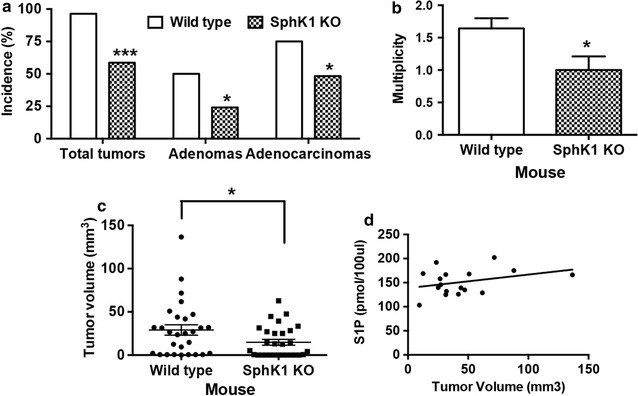
SphK1 KO mice significantly reduce colon carcinogenesis. SphK1 KO mice significantly reduced colon tumor incidence (number of mice with tumors, **a**) including total tumor (adenoma plus adenocarcinoma), adenoma, and adenocarcinoma; tumor multiplicity (number of tumors per mouse, **b**); tumor volume (**c**) as compared to WT mice. Tumor volume is positively correlated to S1P levels in blood in WT mice (**d**). The data is presented as mean ± SEM; *P < 0.05 and ***P < 0.001 vs. wild type

First, we set out to determine if SphK1 deficiency inhibits AOM-induced colon carcinogenesis. SphK1 KO mice were exposed to AOM. All animals were sacrificed at 50 weeks after the start of AOM injections. At the time of the animal sacrifice, 28 SphK1 KO mice and 29 WT mice were alive in this cohort with no obvious abnormalities noted. The final results of colon tumors development are summarized below. We found that 27 WT mice developed colon tumors (96%, 27/28), including adenomas in 14 mice (50%, 14/28) and adenocarcinomas in 21 mice (75%, 21/28); whereas, only 17 SphK1 KO mice developed colon tumors (59%, *P* < 0.001 vs. WT mice), including adenomas in 7 mice (24%, *P* < 0.05 vs. WT mice) and adenocarcinomas in 14 mice (48%, *P* < 0.05 vs. WT mice) (Fig. 1a). In WT mice, 1.6 tumors per mouse were found while only 1.1 tumors per mouse in SphK1 KO mice (*P* < 0.05, Fig. 1b). We found that tumor volume was reduced in SphK1 KO mice as compared to WT mice (14.8 vs. 29.1 mm^3^, *P* < 0.05, Fig. 1c). These results indicate that SphK1 deficiency significantly inhibits the development of colon tumors induced by AOM. We analyzed serum S1P levels in the GEM treated with AOM and noted that serum S1P levels correlated to tumor burden (Fig. 1d).

### SphK1 overexpression in human colon cancer cells enhances tumor growth

**Fig. 2:**
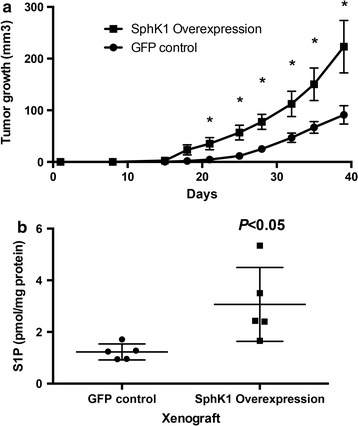
SphK1 overexpression in intestinal epithelial cells significantly increase tumor development induced by AOM. **a** Dox treatment induces SphK1 expression in intestinal epithelial cells. **b** SphK1 overexpression significantly increases tumor multiplicity induced by AOM (*P* < 0.05). The data is presented as mean ± SEM; *P < 0.05 vs. GFP control

We investigated if SphK1 overexpression supports the subcutaneous growth of a human colon cancer xenograft in mice. Using HT-29, human colon cancer cells, we stably transfected with hSphK1-GFP or GFP-empty vector (Fig. 2a). Next, 1 × 10^6^ cells were injected subcutaneously into the left flank of female nude mice (six mice in each). We monitored tumor growth in 6 weeks. We first found tumor formation in SphK1 overexpression mice at 18 days after injection, while tumor formation in GFP control mice at 21 days after injection was observed. Afterwards, mice with SphK1 overexpression cells developed significantly larger tumors than GFP control mice (229.5 mm^3^ vs. 90.9 mm^3^, respectively. *P* < 0.05). As demonstrated in the SphK1 KO mice, serum levels of S1P were higher in tumor bearing animals than controls, Fig. 2b). These results suggest that acquiring SphK1 expression may provide growth advantage to SphK1 expressing tumors.

### SphK1 overexpression in intestinal epithelial cells enhances AOM-induced colon tumor

**Fig. 3:**
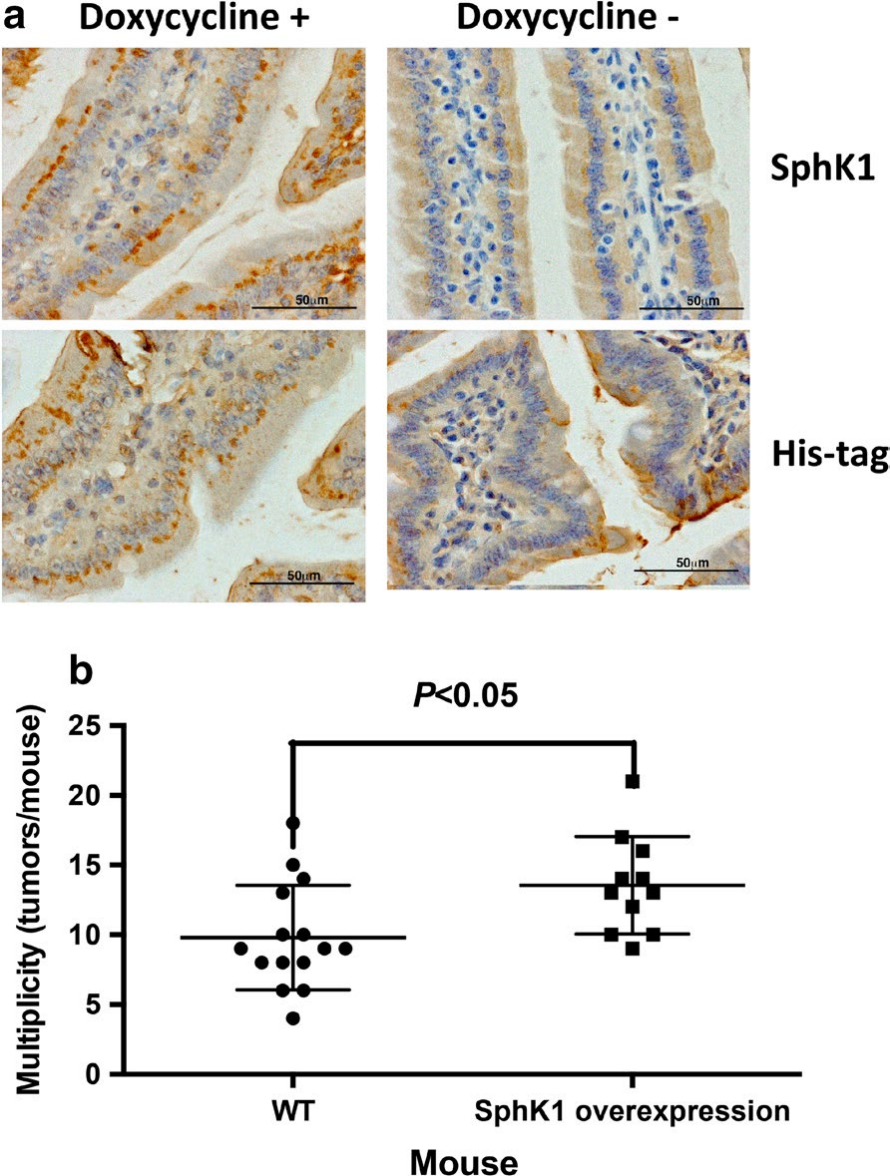
SphK1 overexpression in HT-29, human colon cancer cells, significantly enhance tumor growth. **a** Tumor growth curves in SphK1 overexpression and GFP control HT-29 cells until 38 days and SphK1 overexpression cells significantly enhanced tumor growth during the experiment (*P* < 0.05 after 21 days as compared to GFP control cells). **b** S1P levels in tumors of SphK1 overexpression cells are significantly higher than those in GFP control cells (*P* < 0.05)

Next, we successfully developed double Tg mice that expressed SphK1 in intestinal epithelial cells with doxycycline (Dox) (Fig. 3a). All animals were treated with either injection of AOM at 10 mg/kg body weight or saline once a week for 6 weeks and fed Dox diet during the experiment. We first analyzed effects of FVB/N background and Dox diet on AOM-induced colon carcinogenesis. FVB/N background mice were more susceptible to AOM-induced colon carcinogenesis than C57/BL background. We found that all AOM-treated WT FVB/N mice developed colon cancers (100% vs. 96.4% in incidence) with mean 15 tumors per mouse (multiplicity) that is significantly higher than those in C57BL/6 WT mice (*P* < 0.0001, 15 ± 2.0 vs. 1.64 ± 0.16). Dox treatment significantly reduced AOM-induced colon carcinogenesis in FVB/N mice (9.8 ± 0.97 vs. 15 ± 2.0 in multiplicity, *P* < 0.05), indicating that Dox treatment leading to suppression of SphK1 expression inhibits colon carcinogenesis. Thus, we employed WT FVB/N mice with Dox treatment as a control for double Tg mice with Dox treatment. We found that double Tg mice treated with Dox developed an average of 13.6 tumors per mouse induced by AOM while WT mice with Dox treatment developed only 9.8; indicating that double Tg mice with Dox treatment significantly increased colon tumors as compared to WT with Dox treatment (*P* < 0.05, 13.6 ± 1.06 vs. 9.8 ± 0.97, Fig. 3b). In contrast, we observed no statistically significant differences in tumor incidence or tumor volume in double Tg mice exposed to Dox.

### SphK1 and COX-2 expression in the late stage of human colon cancer

**Fig. 4:**
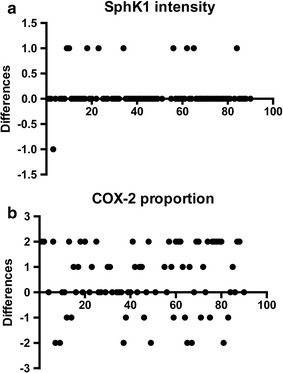
Wilcoxon matched-pairs signed rank test between cancer and adjacent normal tissues. Tissue microarray containing colon cancer tissues and matched adjacent normal tissues from 90 colon adenocarcinoma patients are stained for SphK1 and COX-2. Staining was scored based on the intensity (0–3) and proportion (0–3) and the each score was used for the analysis. The differences between cancer tissues and adjacent normal tissues were calculated and analyzed by Wilcoxon matched-pairs signed rank test. **a** SphK1 intensity, and **b** COX-2 proportion

First, we examined whether SphK1 and COX-2 are overexpressed in cancer tissues as compared to adjacent normal tissues. Since some of tissues were not scored due to quantity not sufficient (QNS), we employed 79 subjects for SphK1 and 78 subjects for COX-2 analysis (Additional file 2). Wilcoxon matched-pairs signed rank test demonstrated that SphK1 intensity was higher in cancer than adjacent normal tissues (cancer vs. adjacent normal, 1.10 ± 0.30 vs. 1.00 ± 0.00, *P* = 0.02, Fig. 4a). There was no statistically significant difference in SphK1 proportion between cancer and adjacent normal tissues (cancer vs. adjacent normal, 2.93 ± 0.30 vs. 3.00 ± 0.00, *P* = 0.1250, Additional file 1: Figure S3A). COX-2 intensity was lower in cancer than adjacent normal tissues (cancer vs. adjacent normal, 1.58 ± 0.50 vs. 1.94 ± 0.23, *P* < 0.0001, Fig. 4b); whereas COX-2 proportion was higher in cancer than adjacent normal tissues (cancer vs. adjacent normal, 2.16 ± 0.91 vs. 1.66 ± 0.81, *P* = 0.0019, Additional file 1: Figure S3B), suggesting that COX-2 is condensed in adjacent normal tissue, while spread out in cancer.

Next, we examined the effect of SphK1 and COX-2 expression on pathophysiology and prognosis colon cancer. Due to QNS, we employed 85 subjects for this analysis. The statistical analysis found that there was no difference in SphK1 and COX-2 intensity and proportion among tumor grades and stages (data not shown), suggesting that SphK1 and COX-2 may play important roles in the early stage of colon carcinogenesis. Since SphK1 staining was very uniform, only 9 of 85 subjects were identified as moderate. Using the Kaplan–Meier survival analysis with the log-rank test, we did not find that SphK1 intensity significantly reduced overall survival (*P* = 0.57, Additional file 1: Figure S4A). In COX-2, 38 subjects were identified as weak, while 47 subjects were identified as moderate. Kaplan–Meier survival analysis did not show that COX-2 intensity significantly reduced overall survival (*P* = 0.32, Additional file 1: Figure S4B).

## Discussion

In this study, we clearly demonstrate that SphK1 KO mice have a reduction in AOM-induced colon carcinogenesis, including tumorigenicity, multiplicity and tumor burden, while forced expression of SphK1 in intestinal epithelial cells exacerbates AOM-induced colon cancer development. Overexpression of SphK1 in human colon cancer xenografts enhances tumor growth. SphK1 and COX-2 intensity tended to reduce overall survival of late stage colon cancer patients.

Previously, our group discussed the role of SphK1 and S1P in dextran sulfate sodium (DSS)-induced colitis, AOM-induced aberrant crypt foci (ACF, colonic preneoplastic lesion) and AOM/DSS-induced inflammation-related colon carcinogenesis [5, 16]. In these studies, it has been suggested that SphK1/S1P pathway regulates colon inflammation and carcinogenesis by mediating COX-2/PGE_2_ pathway. Global gene expression analysis suggested that altered expression in the colonic mucosa in mice treated with AOM/DSS was greater than that found in the mice given AOM alone, suggesting that AOM/DSS model may have distinctive mechanism in addition to AOM alone-induced colon carcinogenesis [17]. On the other hand, histological comparison between these two models demonstrated that both AOM alone and AOM/DSS treatment induce high expression of COX-2 in adenocarcinoma [18].

We also showed that SphK1 overexpression enhances tumor growth in human colon cancer xenografts. When injected subcutaneously in nude mice, although both GFP control and SphK1 overexpressing HT-29 cells produce tumors, the tumor volume in the SphK1 overexpressing was significantly greater than GFP control cells.

Sphingolipid analysis demonstrated that serum S1P level in tumor is higher in SphK1 overexpressed HT-29 cells than in GFP control cells. We have reported that SphK1 and S1P levels were higher in colon cancer induced by AOM than in normal tissue [5]. Previous in vitro experiment demonstrated that S1P stimulates cell proliferation and invasion in colon cancer cell lines [19, 20], and addition of anti-S1P monoclonal antibody reversed all these processes by increasing activation of caspase-3 [20]. Thus, we confirm that SphK1/S1P pathway function as a key regulator of cell growth in colon carcinogenesis.

Several studies including our group demonstrated that SphK1 deficiency inhibits the growth of polyps induced by an adenomatous polyposis coli (APC) mutation, AOM-induced ACF formation and AOM/DSS-induced cancer development [5, 21]. However, no group has previously investigated whether SphK1 enhances colon cancer. Notably, this is the first time in which forced overexpression of SphK1 in intestinal epithelium correlated with accelerated colon cancer development induced by AOM. More importantly, SphK1 overexpression in intestinal epithelium significantly increased tumor multiplicity (tumors per mouse) when compared to WT mice. The result suggests that SphK1 plays an important role in tumorigenicity and that it may be necessary in the early stage of colon carcinogenesis.

Lastly, SphK1 and COX-2 intensity did not significantly reduce overall survival of late stage colon cancer patients. Wilcoxon matched-pairs signed rank test demonstrated that SphK1 intensity and COX-2 proportion were higher in cancer than adjacent normal tissues. However, there was no difference in intensity and proportion of SphK1 and COX-2 among tumor grades and stages. Results may suggest that both SphK1 and COX-2 play important roles in the early stage, but not the late stage of colon cancer.

## Conclusions

The present study shows that the SphK1/S1P pathway plays an important role in colon carcinogenesis and tumor growth, thus inhibition of SphK1 may be an effective strategy for colon cancer chemoprevention. Furthermore, serum SphK1 levels may hold potential in identifying patients with increased colon tumor burden.

## Additional files

**Additional file 1.** Additional table and figures.

**Additional file 2.** Dataset information.

## Abbreviations

SphK: sphingosine kinase
S1P: sphingosine 1-phosphate
GEM: genetically engineered mice
AOM: azoxymethane
PGE_2_: prostaglandin E_2_
KO: knockout
NIDDK: National Institute of Diabetes and Digestive and Kidney Diseases
WT: wild-type
DMEM: Dulbecco’s Modified Eagle’s Medium
FBS: Fetal Bovine Serum
rtTA: reverse tetracycline-regulated trans-activator
MMHCC: Mouse Models of Human Cancers Consortium
PCR: polymerase chain reaction
TMA: tissue microarray
DAB: 3,3′-diaminobenzidine
Dox: doxycycline
QNS: quantity not sufficient
DSS: dextran sulfate sodium
ACF: aberrant crypt foci
APC: adenomatous polyposis coli
