# Supplementary material for: Sphingosine kinase 1 expression enhances colon tumor growth
Source: J Transl Med. 2017 Jun 6;15:120. doi: 10.1186/s12967-017-1220-x (PMC5460497; doi:10.1186/s12967-017-1220-x)
Supplement: Supplementary file 1 — Additional file 1. Additional table and figures. [file 12967_2017_1220_MOESM1_ESM.pdf]

Supplemental table 1. PCR primers for genotyping

	Primer, 5'-3'	
	Forward	Reverse
SphK1 wild type	TGTCACCCATGAACCTGCTGTCCCTGCACA	AGAAGGCACTGGCTCCTCCAGAGGAACAAG
SphK1 mutant	TCGTGCTTTACGGTATCGCCGCTCCCGATT	
tetO-hSphK1 Tg	CGCAAATGGGCGGTAGGCGTG	TAGAAGGCACAGTCGAGG
rtTA Tg	AGGGAAACACCTACTACTGA	ATTCCAAGGGCATCGGTA

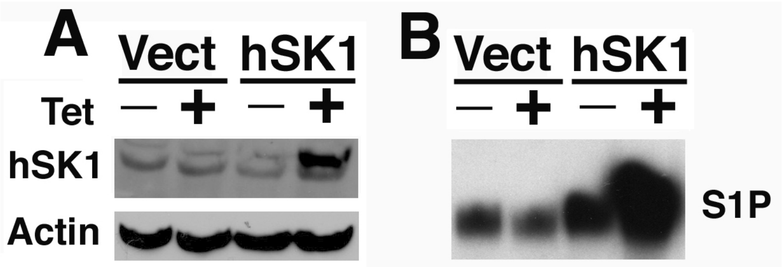

Supplemental Figure S1 - SphK1 overexpression in MCF-7 cells.
A) SphK1 protein level is overexpressed in response to tetracycline treatment. B) SphK1 activity is also increased by tetracycline treatment.

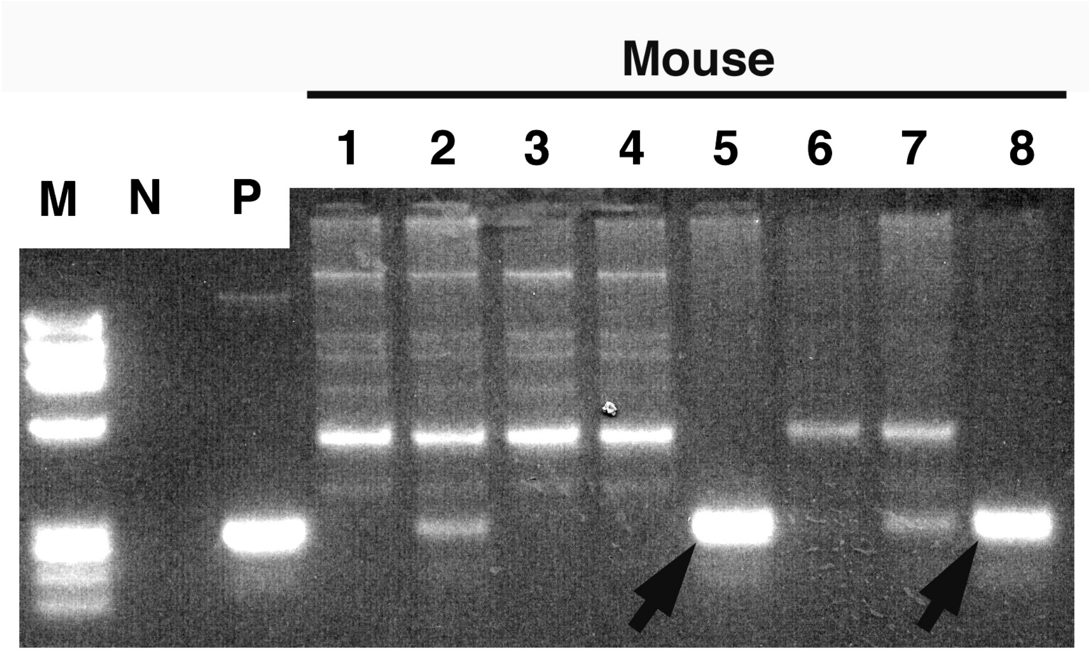

Supplemental Figure S2 - PCR genotyping of tetO-SphK1 Tg mice founders. Allows indicate the founders. M, marker; N, negative control; P, positive control.

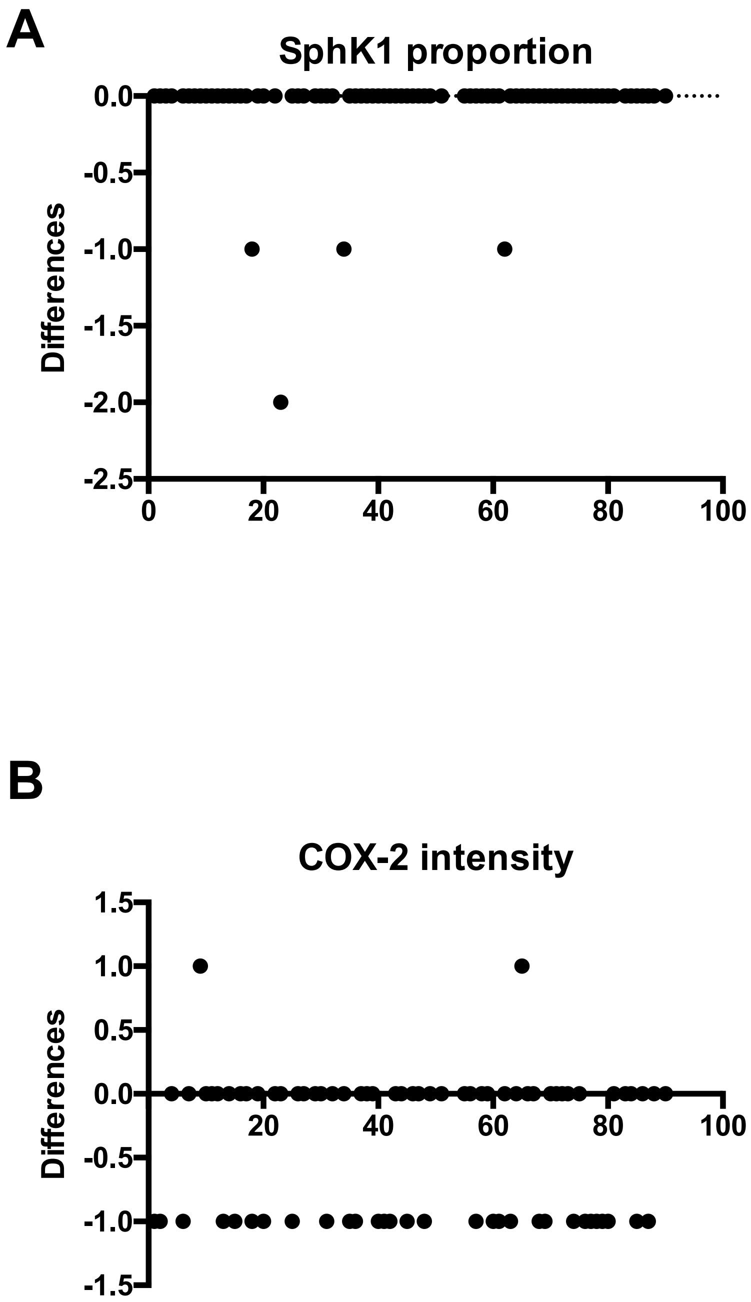

Supplemental Figure S3 - Wilcoxon matched-pairs signed rank test between cancer and adjacent normal tissues. A) SphK1 proportion. B) COX-2 intensity.

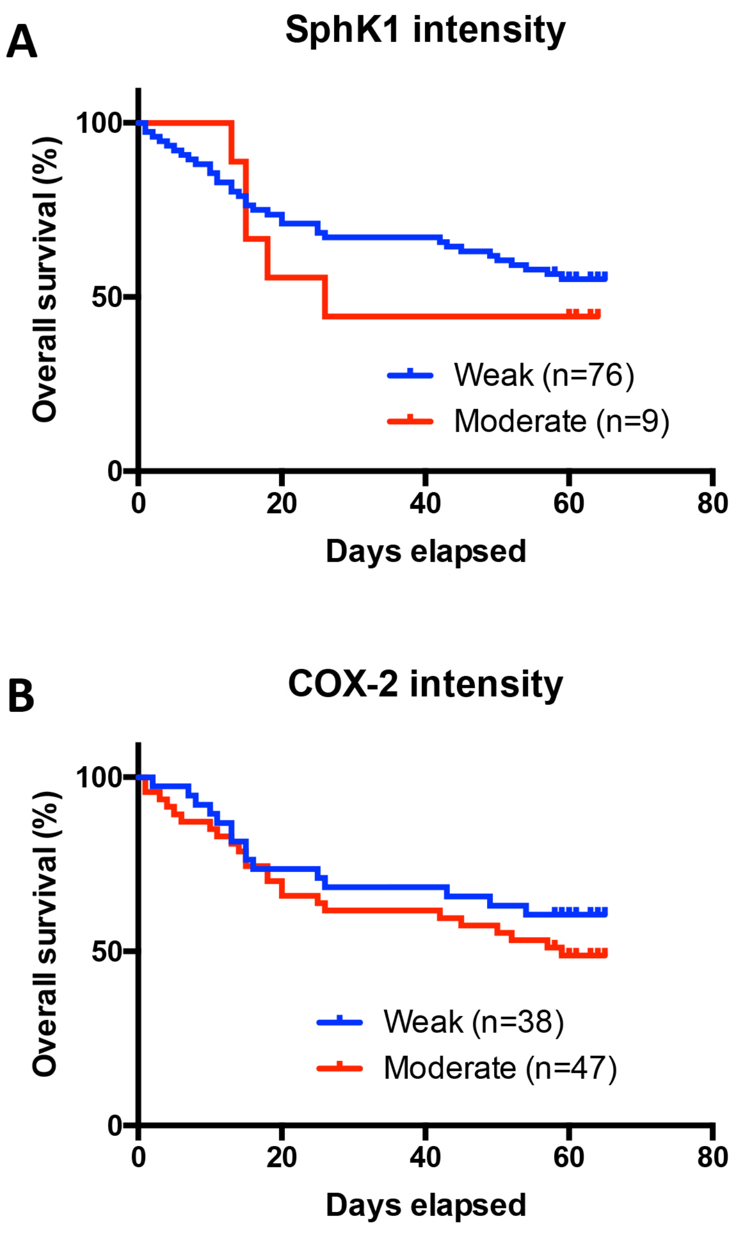

Supplemental Figure S4 - Effects of SphK1 and COX-2 expression on overall survival.
A) SphK1 intensity. B) COX-2 intensity.
